# Protection by Nitric Oxide Donors of Isolated Rat Hearts Is Associated with Activation of Redox Metabolism and Ferritin Accumulation

**DOI:** 10.1371/journal.pone.0159951

**Published:** 2016-07-22

**Authors:** Hilbert Grievink, Galina Zeltcer, Benjamin Drenger, Eduard Berenshtein, Mordechai Chevion

**Affiliations:** 1 Department of Biochemistry and Molecular Biology, Hebrew University of Jerusalem, Jerusalem, Israel; 2 Department of Radiology, Hebrew University—Hadassah Medical Center, Jerusalem, Israel; 3 Anesthesiology and Critical Care Medicine, Hebrew University—Hadassah Medical Center, Jerusalem, Israel; 4 Electron Microscopy Unit, The Core Research Facility, Hebrew University, Jerusalem, Israel; Indiana University School of Medicine, UNITED STATES

## Abstract

Preconditioning (PC) procedures (ischemic or pharmacological) are powerful procedures used for attaining protection against prolonged ischemia and reperfusion (I/R) injury, in a variety of organs, including the heart. The detailed molecular mechanisms underlying the protection by PC are however, complex and only partially understood. Recently, an ‘iron-based mechanism’ (IBM), that includes *de novo* ferritin synthesis and accumulation, was proposed to explain the specific steps in cardioprotection generated by IPC. The current study investigated whether nitric oxide (NO), generated by exogenous NO-donors, could play a role in the observed IBM of cardioprotection by IPC. Therefore, three distinct NO-donors were investigated at different concentrations (1–10 μM): sodium nitroprusside (SNP), 3-morpholinosydnonimine (SIN-1) and S-nitroso-N-acetylpenicillamine (SNAP). Isolated rat hearts were retrogradely perfused using the Langendorff configuration and subjected to prolonged ischemia and reperfusion with or without pretreatment by NO-donors. Hemodynamic parameters, infarct sizes and proteins of the methionine-centered redox cycle (MCRC) were analyzed, as well as cytosolic aconitase (CA) activity and ferritin protein levels. All NO-donors had significant effects on proteins involved in the MCRC system. Nonetheless, pretreatment with 10 μM SNAP was found to evoke the strongest effects on Msr activity, thioredoxin and thioredoxin reductase protein levels. These effects were accompanied with a significant reduction in infarct size, increased CA activity, and ferritin accumulation. Conversely, pretreatment with 2 μM SIN-1 increased infarct size and was associated with slightly lower ferritin protein levels. In conclusion, the abovementioned findings indicate that NO, depending on its bio-active redox form, can regulate iron metabolism and plays a role in the IBM of cardioprotection against reperfusion injury.

## Introduction

Nitric oxide (NO) is a highly reactive diatomic molecule produced in various tissues, including the myocardium, by a family of enzymes called NO-synthases (NOS). NOS catalyze the stepwise conversion of L-arginine and O_2_, to L-citrulline and NO [1, 2]. Two of the three NOS isoforms, endothelial (eNOS) and neuronal (nNOS), are expressed constitutively also in the heart. Conversely, the inducible form (iNOS) releases NO as a defense against stress (e.g., inflammation). NO is an important signaling molecule [3, 4] and plays key roles in modulating cardiomyocyte function [5] and cardioprotection [6–10]. On the molecular level, NO has been associated with the activation of various cell survival pathways and antiapoptotic genes [11]. The biological effects of NO depend strongly on its concentration, the cellular redox state, the presence of reactive oxygen species (ROS), and the subsequent identity of its bio-active redox forms.

Redox related species of NO include; NO^•^, which can modulate iron (Fe)-containing proteins by direct coordination to iron-centers of heme [12–14] and non-heme (iron-sulfur; Fe-S) proteins [13, 15]. NO^•^ readily reacts with O_2_^-.^ to produce peroxynitrite (ONOO^−^) [16]. ONOO^−^ can, amongst other reactions, cause nitration of tyrosine, including tyrosine residues in proteins [17, 18] and affect their function and stability [19, 20]. The second important species of NO, is the nitrosonium ion (NO^+^), which can nitrosylate thiol groups of proteins, a modification that may have important regulatory functions [21–23]. NO^+^ has a short half-life (≈10^−10^ s) in solution at physiological pH [24], and binds rapidly to thiol groups resulting in–SNO-containing compounds that maintain a ‘nitrosonium character’. The subsequent transfer of NO^+^ to other thiols can lead to alterations in protein function, stability and location [22, 25–28].

**Fig 1:**
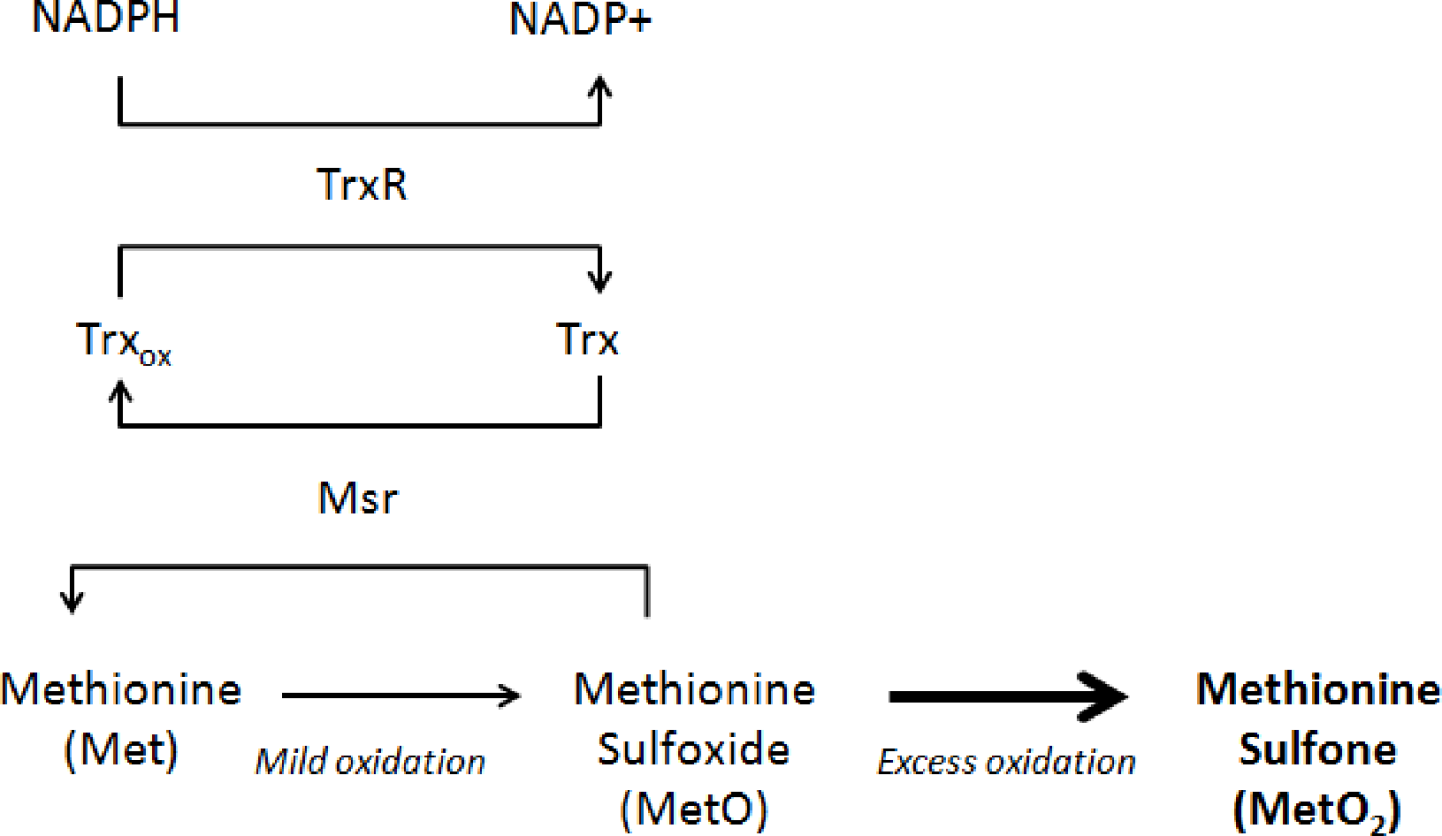
The Methionine-Centered Redox Cycle. The formation of methionine sulfoxide (MetO) can result from the oxidation of free methionine, or a methionyl residue of a protein. Additional oxidation will generate methionine sulfone (MetO_2_), a product that is almost irreversible in biological systems, and can cause protein denaturation. MetO can be reduced by methionine sulfoxide reductases (MsrA or MsrB isoform), through thioredoxin (Trx). Thioredoxin reductase (TrxR) regenerates the oxidized Trx (Trx_ox_) via critical components of the cellular redox system, NADP/NADP(H) [32].

Methionine residues are among the most susceptible to oxidation by ROS [29]. The Methionine-Centered Redox Cycle (MCRC) is an enzymatic system that catalyzes the reduction of, free and protein-bound, oxidized methionine (MetO). For its action it utilizes methionine sulfoxide reductases (Msr), thioredoxin (Trx), thioredoxin reductase (TrxR), and NADPH (Fig 1). Malfunction of the MCRC system can lead to cellular changes resulting in compromised antioxidant defense, enhanced age-associated diseases involving neurodegeneration, and shorter life span [30, 31].

Recently, we have showed that ischemic preconditioning (IPC) led to an ‘iron signal’, accumulation of cellular ferritin, and the activation of an ‘iron-based mechanism’ (IBM) of myocardial protection against ischemia and reperfusion (I/R) injury [33]. The source of iron for the iron signal was found to originate from the proteosomal degradation of ferritin [34]. Ferritin, the major iron storage and detoxifying protein, chelates the harmful redox active iron that is released during ischemia [35–37]. The expression of ferritin is post-transcriptionally regulated by the iron-regulatory proteins (IRPs), IRP1 and IRP2 [38]. When intracellular iron is low, both IRP1 and IRP2 bind with high affinity to the iron-responsive element (IRE) within the ferritin mRNA, inhibiting its translation. When iron is abundant, IRP1 combines with it and dissociates from the IRE, allowing for the renewal of ferritin mRNA translation. Under these conditions IRP1 exhibits cytosolic aconitase (CA) activity. CA is part of the metabolic pathway that converts citrate to iso-citrate and then to α-ketoglutarate. The latter reduces NADP+ to NADPH. NADPH is an essential cofactor in the MCRC, glutathione metabolism and lipid and cholesterol biosynthesis [39, 40].

In the current study the effects of three distinct NO-donors were investigated on hearts submitted to prolonged ischemia and reperfusion; sodium nitroprusside (SNP), 3-morpholinosydnonimine (SIN-1) and S-nitroso-N-acetylpenicillamine (SNAP). The effects of the different NO-donors on myocardial infarct size, hemodynamic function, as well as the MCRC system and iron homeostasis were assessed. The findings presented here, provide insight into the mechanistic pathways underlying the cardioprotective effects generated by NO, and suggest a role of NO in the IBM of myocardial protection.

## Materials and Methods

### Animals

Male Sprague-Dawley rats weighing 300–350 g were fed a regular laboratory diet and had free access to food and water. The rats were acclimated to the local animal facility for at least four days prior to use in an experiment. During this time, the physical conditions of the rats were monitored regularly. No animals became ill or died prior to the experimental end point. All the experimental protocols were approved by the ‘Institutional Animal Care and Use Committee’ of the Hebrew University of Jerusalem, conforming to the Guide for the Care and Use of Laboratory Animals published by the U.S. National Institutes of Health (NIH Publication No. 85–23, revised 1996).

### Perfusion technique

Rats were injected intraperitoneally (IP) with sodium heparin (500 units) and 20 min later with sodium pentobarbital (60mg/kg). Confirmation of deep anesthesia and an unresponsiveness to pain stimuli was confirmed by a negative paw withdraw reflex. Hearts were then rapidly removed and placed in heparinized ice-cold saline. Each heart was then cannulated via the aorta and retrogradely perfused at a constant perfusion pressure of a 90 cm water column. The standard perfusate consisted of modified Krebs-Henseleit (KH) buffer containing (mM) NaCl, 118; KCl, 4.5; KH_2_PO_4_ 1.3; CaCl_2_, 2.5; MgSO_4_, 1.2; NaHCO_3_, 25 and glucose, 11.1. The perfusion buffer was gassed with 95% O_2_ and 5% CO_2_ and pH was maintained at 7.4. The NO-donors (SNP, SNAP and SIN-1) were added at the indicated time points and concentrations along the perfusion protocol. Hearts were kept in a thermostated glass cell, at a constant temperature of 37.0°C± 0.1. Upon the initiation of the experiment, a small latex balloon-tipped catheter was inserted into the left ventricle through an incision on the left atrium. The balloon was connected via a pressure transducer to a recording system that allowed monitoring of the peak systolic pressure (PSP), the end diastolic pressure (EDP), the developed pressure (DP) = PSP–EDP, the positive and negative derivatives of DP (+dp/dt and–dp/dt), and the heart rate (HR). The work index (WI) was calculated according to WI = DP x heart rate.

### Experimental protocol

**Fig 2:**
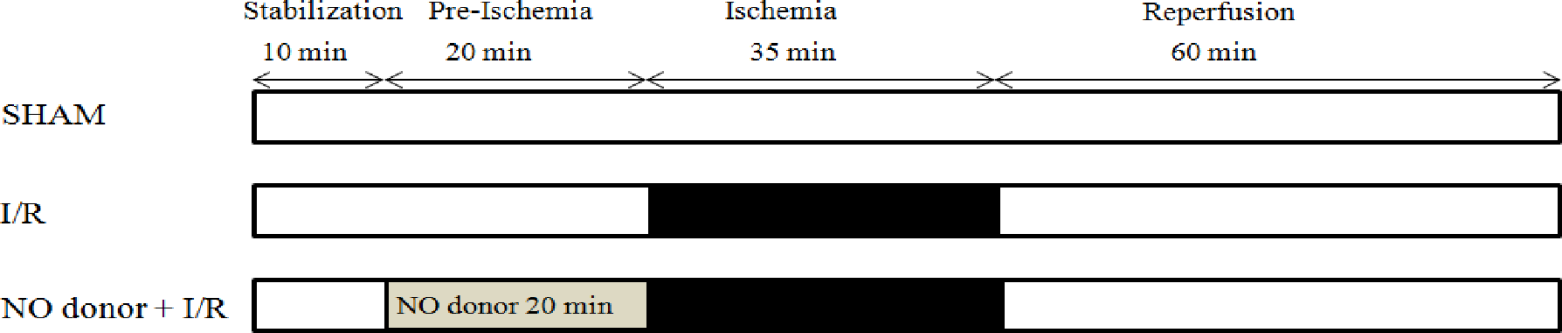
Experimental protocols.

Fig 2 represents the experimental designs and perfusion protocols. Each experimental group contained 8 hearts, which were used for biochemical analyses. Infarct size analyses were conducted on 4–6 additional hearts. The combined hemodynamic data were used for functional analyses. A typical experiment was started by perfusion for 10 min with KH-solution in order to stabilize cardiac function and to determine the basal hemodynamic parameters. The heart was then subjected to an additional 20 min perfusion with KH-buffer (without or with the addition of a NO-donor). Subsequently, the heart was subjected to 35 min of no-flow global ischemia, followed by 60 min of reperfusion.

### Infarct size analyses

At the end of reperfusion, hearts were frozen for 15 min at -20°C before slicing into five transverse slices, parallel to the atrioventricular groove. After removing right ventricular and atrial tissue, heart slices were incubated for 30 min in a 1% solution of triphenyltetrazolium chloride at 37°C. This allowed differentiation of the infarcted (pale) from viable (bright red) myocardial area [41]. The size of the infracted tissue was digitally photographed with a Nikon Coolpix 5000 camera and quantified with IMAGE J 1.32 (NIH, USA) software. Determination of the area of infarction was performed by a blinded investigator.

### Methionine sulfoxide reductase activity

Measurements of the Msr activities were carried out by incubating the heart homogenates with dabsyl-methionine sulfoxide for 30 min at 37°C. Subsequently, analysis of the reduced product (dabsyl methionine) was conducted by a HPLC-photometric detection at 436 nm [29, 42]. The reaction mixtures contained 200 μM Dabsyl-MetO (the substrate)/ 20 mM Tris⋅HCl (pH 7.5)/10 mM MgCl_2_/30 mM KCl/20mM dithiotreitol (DTT), and ~100μg protein; total volume of 100μL. The reactions were stopped by adding 100μL of acetonitrile. The samples were then spun down and the protein fractions were discarded. In the chromatographic assays, the samples were run on a 150 mm 3μm C-18 column, using a gradient (A to B). A = 19g of sodium acetate, pH 6.0 plus 0.5ml of triethylamine, in one liter of solution; B = acetonitrile (pure). The substrate, dabsyl-Met(O) was prepared according to Moskowitz et al. [42]. Activities were given as pmol/mg protein/min.

### Western blot analysis

Quantifications of Trx and TrxR proteins were conducted by Western blot analyses as previously described, with minor modifications [32, 43]. Briefly, equal amounts of protein (5 μg) were separated by SDS-PAGE and transferred to a nitrocellulose membrane at 250 mA for 90 min. Membranes were blocked at 4°C overnight with 5% dry skim milk in 0.05 M Tris-buffered saline pH 7.6, containing 0.05% Tween-20 (TBS-T). Subsequently, the membranes were incubated with Trx or TrxR primary antibodies, which were generously provided by Dr. S.G. Rhee (Ewha Women University, Seoul, Korea). After washing with TBS-T, the membranes were incubated for 1h at room temperature with HRP-labeled goat anti-rabbit IgG (Jackson ImmunoResearch Laboratories Inc. West Grove, PA, USA). Next, the membranes were washed with TBS-T and developed using light sensitive film (Amersham Biosciences, Little Chalfont, Buckinghamshire, UK) and the chemiluminescence detection kit for HRP (EZ-ECL; Biological Industries, Beit-Haemek, Israel) according to manufacturer's instructions. Band intensities were quantified using IMAGE J 1.32 software (NIH, USA).

### Cytosolic aconitase activity

At the end of the experiments, one half of the left ventricle was homogenized in a buffer containing Tris-HCl, 50 mM; cysteine, 1 mM; sodium citrate, 1 mM; and MnCl_2_, 0.5 mM; pH 7.6. To rule out contamination with mitochondrial aconitase, digitonin (0.02%) was added to the buffer before homogenization, in order to preserve the inner mitochondrial membrane. Homogenates were centrifuged at 1800 g for 8 min. Supernatants were re-centrifuged at 11,000 g for 20 min at 4°C; the pellets containing the mitochondrial fraction were discarded; the supernatants representing the cytosol were kept on ice until analyzed. The cytosolic fraction was checked and cleared of mitochondrial proteins by western blot. The aconitase activity in the reaction mixture was measured in duplicates by a coupled assay using isocitrate dehydrogenase and NADP^+^, which was reduced to NADPH in the course of the reaction. Briefly, 135 μL of the reaction mixture (90 μL sample, 35 μL (2 unit/mL) isocitrate dehydrogenase, 10 μL (0.2 mM NADP^+^) was added to 865 μL assay buffer consisting of 100 mM Tris-HCl, 30 mM sodium citrate and 0.5 mM MnCl_2_, pH 7.4. The rate of NADPH^+^ reduction was measured photometrically at 340 nm over 5–10 min, at 37°C. Protein content of heart extracts was assessed using Bio-Rad reagent and bovine serum albumin as a standard.

### Total protein content

Total cytosolic protein content was determined using the BCA (bicinchoninic acid) Protein Assay Kit (Pierce, Rockford, IL, USA).

### Heart ferritin levels

Heart ferritin levels were determined in the cytosolic fractions using a previously described ELISA-based method [36].

### Iron content in a ferritin molecule

After immuno-precipitation, ferritin was dissolved in nitric acid and iron content was determined by Zeeman atomic absorption spectroscopy [36] or spectrophotometrically using batho-phenanthroline bi-sulphonate (BPS) [44]. Subsequently the average number of iron atom per ferritin molecule was calculated.

### Statistical data analyses

Data are presented as Mean ± SEM. Statistical analyses between values of the same group at various stages of the protocol were performed by one-way analyses of variance (ANOVA). Between groups comparisons were made for each time point using a one-way ANOVA followed by the Dunnett’s post-hoc test, where appropriate. Changes were considered statistically significant when p<0.05.

## Results

### Hemodynamics

Table 1 represents the hemodynamic data and recoveries of the hearts after exposure to the different perfusion protocols. There were no significant differences in baseline values for HR, EDP and left ventricular DP or its derivatives; +dp/dt and–dp/dt, in any of the experimental groups. SHAM treated hearts maintained ~90% of their function compared to baseline, as depicted by the hemodynamic parameters in Table 1. In addition, EDP values remained at baseline levels in the SHAM group. These values were significantly better than hearts subjected to I/R alone. Hearts subjected to I/R alone, lost 60% of their basal WI capacity, by the completion of the reperfusion phase. DP recovered to 43% of the pre-ischemic value, whereas HR recovered fully. The EDP at the end of the reperfusion, was increased significantly in the I/R group. Treatment with the different NO-donors for 20 min, before the onset of ischemia, did not have any significant effects on hemodynamic recovery in any of the experimental groups. Nonetheless, hearts treated with 10 μM SNAP and 100 μM SNP displayed slightly lower EDP values compared to hearts exposed to I/R alone. In contrast, there is a trend towards lower recoveries of WI capacities after treatment 2 μM SIN-1, 10 μM SIN-1 and 2 μM SNAP. A concentration dependent effect can be detected after perfusion with SNAP. The hemodynamic recoveries (%) of hearts perfused with 10 μM SNAP are consistently better compared to hearts perfused with 2 μM SNAP. Such an effect was not observed between the groups perfused with 2 μM or 10 μM SIN-1.

**Table 1:** Hemodynamic parameters of the rat hearts after exposure to the different experimental protocols.

**Protocol**	HR_0_ (min^-1^)	DP_0_ (mm Hg)	**HR (%)**	**DP (%)**	**WI (%)**	EDP_125_ (mm Hg)
**SHAM**	261 ± 8	83 ± 3	104 ± 5	93 ± 2*	96 ±6*	5 ± 2*
**I/R**	288 ± 8	101 ± 6	91 ± 9	43 ± 5	40 ± 6	44 ± 4
**100** μ**M SNP + I/R**	273 ± 12	93 ± 5	84 ± 5	46 ± 6	44 ± 7	37 ± 5
**2** μ**M SIN-1 + I/R**	283 ± 8	89 ± 6	72 ± 5	39 ± 6	29 ± 6	47 ± 8
**10** μ**M SIN-1 + I/R**	253 ± 14	98 ± 10	55 ± 10	37 ± 7	23 ± 9	43 ± 5
**2** μ**M SNAP + I/R**	268 ± 13	97 ± 6	64 ± 8	31 ± 5	22 ± 7	47 ± 5
**10** μ**M SNAP + I/R**	272 ± 11	97 ± 9	93 ± 8	47 ± 5	42 ± 5	35 ± 9

The hemodynamic recoveries (%) of the heart rate (HR), developed pressure (DP) and the work index (WI), at the completion of each of the protocols was compared to the pre-ischemic values. The following additional abbreviations were used: I/R–ischemia/reperfusion; EDP_125_ –end diastolic pressure at the completion of the experiment (125^th^ min); WI–work index; DP_0_ and HR_0_– developed pressure and heart rate at the stabilization phase, respectively. Data are presented as Mean ± SEM.

* p < 0.01 versus I/R.

### Infarct size

**Fig 3:**
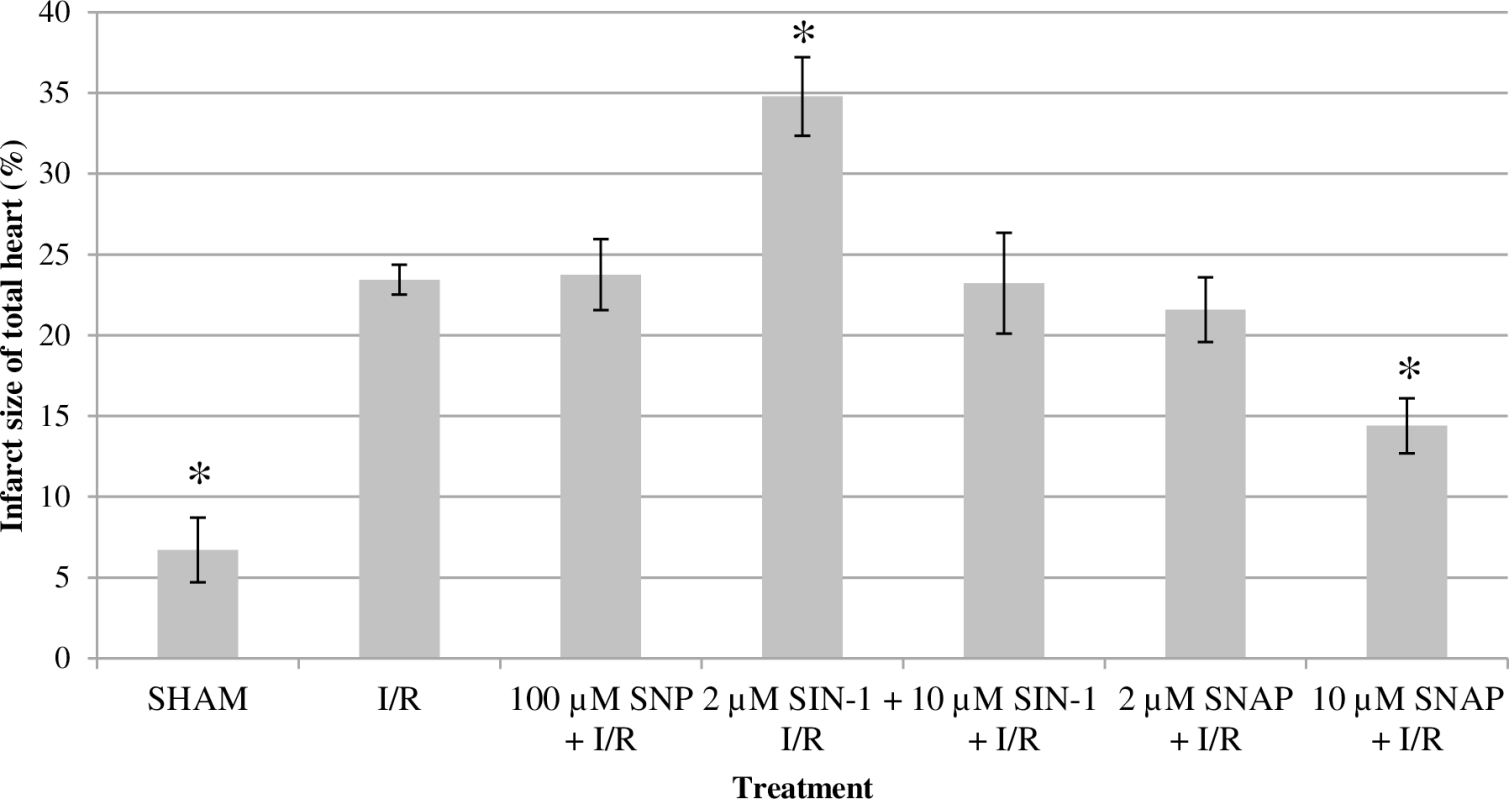
Infarct sizes of the rat hearts after exposure to the different experimental protocols. Data are presented as Mean ± SEM.* p < 0.05 versus I/R.

Infarct size was (23.4 ± 0.9) % in hearts exposed to I/R alone (Fig 3). SHAM treated animals had significantly lower infarct sizes (6.7 ± 2.0%; p < 0.05 vs. I/R). Treatment with 10 μM SNAP for 20 min, before prolonged ischemia and reperfusion, significantly reduced infarct size to (14.4 ± 1.7%; p<0.05 vs. I/R). Treatment with 2 μM SNAP (21.6 ± 2.0) %, 100 μM SNP (23.7 ± 2.2) % or 10 μM SIN-1 (23.3 ± 3.1) % had no infarct sparing effect. 2 μM SIN-1 was found to significantly increase infarct size (34.8 ± 2.4%; p < 0.05) compared to I/R alone.

### Methionine-Centered Redox Cycle

Msr activity (expressed as: pmol/mg protein/min) was not significantly different at the end of the reperfusion between the SHAM (3650 ± 433) and I/R groups (3656 ± 605) (Table 2). Perfusion with the NO-donors: 100 μM SNP (6681 ± 1046), 2 μM SNAP (7575 ± 543) or 10 μM SNAP (8521 ± 576) significantly increased Msr activity compared to I/R alone (p < 0.01), as measured at the end of reperfusion. Concentration dependent effects on Msr activities can be observed after perfusions with 2 μM and 10 μM SNAP and SIN-1. Perfusion with SIN-1 did not significantly increase Msr activity compared to the I/R group. Furthermore, hearts perfused with 2 μM SIN-1 generated the lowest Msr activities (4591 ± 304). These findings are in line with the observed increase in infarct sizes in the 2 μM SIN-1 perfused hearts. As perfusion with 10 μM SNAP resulted in a significantly reduction in infarct size (Fig 3), additional time points along this perfusion protocol were investigated. 10 μM SNAP had a profound stimulatory effect on the Msr activity at each time point along the protocol. Immediately after completion of the perfusion with SNAP (Pre-Ischemia), the Msr activity was approximately two fold (11277 ± 238; p < 0.01) higher compared to untreated hearts (5833 ± 711). This higher Msr activity was maintained during the ischemic period (11820 ± 647; p < 0.01 vs. I/R).

**Table 2:** Activities of methionine sulfoxide reductase.

**Protocol**	**Msr activity (pmol/mg protein/min)**		
	**Pre-Ischemia**	**End of Ischemia**	**End of reperfusion**
**SHAM**	5833 ± 711	-	3650 ± 433
**I/R**	5833 ± 711	5409 ± 399	3656 ± 605
**100** μ**M SNP + I/R**	-	-	6881 ± 1046*
**2** μ**M SIN-1 + I/R**	-	-	4591 ± 304
**10** μ**M SIN-1 + I/R**	-	-	6061 ± 429
**2** μ**M SNAP + I/R**	-	-	7575 ± 543*
**10** μ**M SNAP + I/R**	11277 ± 238*	11820 ± 647*	8521 ± 576*

Data are presented as Mean ± SEM and indicate the activity of Msr as measured at different time points along the perfusion protocols.

* p < 0.01 versus I/R.

**Fig 4:**
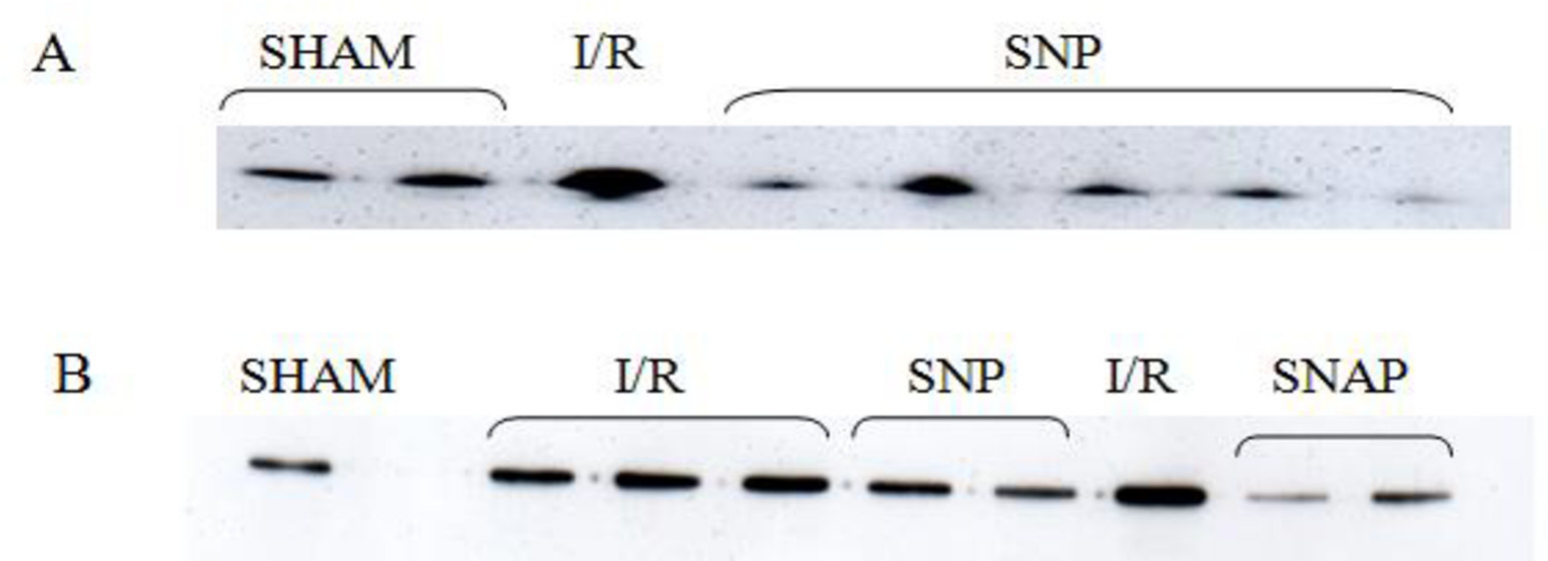
Western blots of cytosolic Thioredoxin and Thioredoxin reductase. Representative western blots used for the protein estimations of cytosolic thioredoxin (A) and cytosolic thioredoxin reductase (B).

The proteins levels of Trx and TrxR were determined by western blot analyses and are shown in Fig 4 and Table 3. Heart subjected to I/R (3.66 ± 0.36 AU.), showed an 8-fold increase in Trx levels compared to SHAM treated hearts (0.46 ± 0.16 AU.; p < 0.01 vs. I/R). All NO-donors prevented this increase and had significantly lower Trx proteins levels compared to I/R treated hearts (p < 0.01). Among the NO-donors, hearts perfused with 2 μM SIN-1 had the highest Trx protein levels at the end of reperfusion (Table 3). The Pre-Ischemic protein level of Trx, was found to be significantly higher after perfusion with 10 μM SNAP (2.47 ± 0.17 AU.; p < 0.01) compared to untreated hearts (0.42 ± 0.05 AU.) During the subsequent I/R the Trx protein level dropped to (0.66 ± 0.06 AU., p < 0.01 vs. I/R). Hearts perfused with 10 μM SNAP also significantly lowered TrxR protein levels (2.42 ± 0.16 AU.) levels at the end of reperfusion compared to hearts treated by I/R alone (4.94 ± 0.87 AU.; p< 0.01). None of the other treatment groups showed significant different TrxR proteins levels compared to I/R treated hearts (Table 3).

**Table 3:** Thioredoxin and thioredoxin-reductase protein levels.

**Protocol**	**Trx(AU)**		**TrxR (AU)**
	**Pre-Ischemia**	**End of reperfusion**	**End of reperfusion**
**SHAM**	0.42 ± 0.05	0.46 ± 0.16*	3.09 ± 0.25
**I/R**	0.42 ± 0.05	3.66 ± 0.36	4.94 ± 0.87
**100** μ**M SNP + I/R**	-	0.32 ± 0.07*	3.17 ± 0.50
**2** μ**M SIN-1 + I/R**	-	1.60 ± 0.52*	6.06 ± 1.29
**10** μ**M SIN-1 + I/R**	-	0.63 ± 0.01*	5.24 ± 0.23
**2** μ**M SNAP + I/R**	-	0.68 ± 0.10*	2.93 ± 1.29
**10** μ**M SNAP + I/R**	2.47 ± 0.17*	0.66 ± 0.06*	2.42 ± 0.16*

Data are presented as Mean ± SEM and indicate the protein levels of thioredoxin 1(Trx) and thioredoxin reductase 1 (TrxR) as measured at various time points along the perfusion protocols.

* p < 0.01 versus I/R.

### Cytosolic aconitase activity

CA activities (expressed as: μM/ (min x mg protein); Table 4) were significantly lower in SHAM (7.1 ± 1.5; p<0.01) compared to I/R (12.7 ± 1.1) perfused hearts. Both 10 μM SIN-1 (22.8 ± 0.6; p < 0.05 vs. I/R) and 10 μM SNAP perfused hearts (21.7 ± 1.5; p < 0.05) significantly increased CA activities compared to I/R alone (12.7 ± 1.1).

**Table 4:** Activity of cytosolic aconitase.

**Protocol**	CA Activity (μM/ (min x mg protein))
**SHAM**	7.1 ± 1.5*
**I/R**	12.7 ± 1.1
**100** μ**M SNP + I/R**	17.9 ± 2.6
**2** μ**M SIN-1 + I/R**	19.2 ± 1.5
**10** μ**M SIN-1 + I/R**	22.8 ± 0.6*
**2** μ**M SNAP + I/R**	19.1 ± 2.0
**10** μ**M SNAP + I/R**	21.7 ± 1.5*

Data are presented as Mean ± SEM and indicate the CA activity as measured at the end of reperfusion.

* p < 0.05 versus I/R.

### Heart ferritin levels

Heart ferritin levels (expressed as: μg Ft/mg protein) were measured along the different time points of the various perfusion protocols and are depicted in Table 5. Analyses of heart ferritin levels at all time points were conducted only for the SHAM, I/R and 10 μM SNAP groups (Table 5). After perfusion with 10 μM SNAP (Pre-Ischemia) ferritin levels increased (0.308 ± 0.008), without reaching statistical significance compared to control (0.225 ± 0.047). At the end of ischemia, ferritin levels in 10 μM SNAP (0.358 ± 0.060) perfused hearts were significantly higher compared to I/R (0.140 ± 0.014; p < 0.01) and SHAM (0.218 ± 0.016; p < 0.05). The ferritin levels in 10 μM SNAP (0.271 ± 0.017; p < 0.01) perfused hearts, remained significantly elevated until the end of reperfusion compared to I/R (0.192 ± 0.016) and SHAM (0.181 ± 0.027). No statistical difference was detected between the I/R and SHAM groups at any of the time points. Ferritin levels, at the end of reperfusion, were also determined for hearts perfused with 100 μM SNP and 2μM SIN-1. The ferritin levels in both groups were lower compared to I/R but did not reach statistical significance (Table 5).

**Table 5:** Ferritin and iron content at various times of the perfusion protocol.

**Protocol**	**Time points**	Ferritin (μg Ft/mg protein)	N_Fe_
**SHAM**	**Stabilization**	0.209 ± 0.037	2250 ± 80
	**Pre-Ischemia**	0.225 ± 0.047	2250 ± 80
	**End of Ischemia**	0.218 ± 0.016	N.D.
	**End of reperfusion**	0.181 ± 0.027	1351 ± 112
**I/R**	**Stabilization**	0.209 ± 0.037	2250 ± 80
	**Pre-Ischemia**	0.225 ± 0.047	2250 ± 80
	**End of Ischemia**	0.140 ± 0.014	3169 ± 4
	**End of reperfusion**	0.192 ± 0.016	1335 ± 79
**10** μ**M SNAP**	**Stabilization**	0.209 ± 0.037	2250 ± 80
	**Pre-Ischemia**	0.308 ± 0.008	1280 ± 14
	**End of Ischemia**	0.358 ± 0.060 *#	1309 ±14
	**End of reperfusion**	0.271 ± 0.017 *	1313 ± 64
**100** μ**M SNP**	**End of reperfusion**	0.138 ± 0.013	1942 ± 90
**2** μ**M SIN-1**	**End of reperfusion**	0.148 ± 0.037	3156 ± 79

Date are presented as Mean ± SEM and indicate the heart ferritin levels and the average number of iron atoms per ferritin molecule (N_Fe_), at various time points along the perfusion protocols. Complete analyses of heart Ft levels was only conducted for the I/R, SHAM and 10 μM SNAP protocols. Iron content was determined in duplicate. Therefore no statistical analyses were conducted on the N_Fe_ ratios for the different groups N.D.- non-determined.

* p < 0.01 versus I/R and SHAM.

^#^ p < 0.05 vs. SHAM.

### Iron atoms per ferritin molecule

N_Fe_ -the average number of iron atoms per ferritin molecule—were drastically elevated at the end of ischemia in I/R (3169 ± 4) N_Fe_, compared to 10 μM SNAP (1280 ± 14) N_Fe_ perfused hearts (Table 5). At the end of reperfusion, the N_Fe_ ratios were similar among the I/R, SHAM and 10 μM SNAP perfused hearts. In the 2 μM SIN-1 perfused hearts, a 2.4 fold increase in the Fe/Ft ratio was detected at the end of the reperfusion (3156 ± 79) N_Fe_ compared to I/R (1335 ± 79) N_Fe_, SNAP (1313 ± 64) N_Fe_ or SHAM (1351 ± 112) N_Fe_. 100 μM SNP perfusion hearts also had a slightly increased ratio (1942 ± 90) N_Fe_ compared to the other groups at the end of reperfusion.

## Discussion

IPC is a well-known procedure which can generate a transient protection of the heart against injury associated with prolonged ischemia and reperfusion. Recently an IBM for cardioprotection by IPC was proposed [33]. The IBM involves the generation of an iron signal, through activation of the proteasome, which results in the accumulation of ferritin [33, 34]. Ferritin can chelate the considerable amounts of mobilized cellular labile iron, during the subsequent prolonged ischemia and reperfusion, and so generate cardioprotection by IPC. Numerous studies have suggested a role of NO in IPC [6–10, 45]. The regulatory capacities of NO are however complex and dependent on its concentration, the cellular redox state, the presence of ROS and the subsequent identity of its bio-active redox forms [23, 27, 46]. The current study investigated a possible link between NO, cardioprotection and the IBM.

Three distinct NO-donors, SNP, SNAP and SIN-1, were employed at various concentrations previously shown to evoke cardioprotection. SNP is known to generate NO^+^, SNAP potentially generates both NO^+^ and NO^.^, whereas SIN-1 generates NO^.^ and O_2_^-.^, which react to form ONOO^−^ [24, 47]. In contrast to the cardioprotective effects of ONOO^−^, several studies have also reported apparent cardiotoxic effects of ONOO^−^ [48]. It has been suggested that this discrepancy could be dependent on the crystalloid buffer or biological (i.e. blood) environment [48–50]. In the current study, infarcts size analyses revealed a dose dependent and NO-donor dependent effect on myocardial infarct size. A significantly reduction in infarct size was only observed in Langendorff perfused isolated rat hearts after a 20 min perfusion with 10 μM SNAP prior to the onset of global ischemia and reperfusion (Fig 3). These results support prior observations on the infarcts sparing ability of SNAP treatment before ischemia and reperfusion [8, 51]. Unlike previous findings presented by Nakano, *et al*., 2 μM SNAP was not found to reduce infarct size [8]. None of the other NO-donors used in the current study generated an infarct sparing effect. Moreover, perfusion with 2 μM SIN-1 prior to prolonged ischemia and reperfusion was found to increase infarct size compared to hearts exposed to I/R alone. These findings are in line with previous studies in which peroxynitrite was largely found to generate a deleterious effect in a crystalloid buffer environment [49, 50, 52]. The observations that perfusion with 2 μM SIN-1 significantly increased infarct size, whereas 10 μM SIN-1 did not, suggests that the deleterious effects of ONOO^−^ in a crystalloid buffer environment are concentration dependent [49]. One can hypothesize, that increasing concentrations of NO^.^, O_2_^-^ and/or ONOO^−^ differently affect endogenous pathways and mechanisms (e.g. NOS activation, nitrosylations). Subsequent differences in NO^.^ and O_2_^-^ stoichiometry, could alleviate some of the deleterious effects of ONOO^−^ [49, 50]. Further investigations are necessary to validate such hypotheses.

In previous studies, 100 μM SNP and 10 μM SIN-1 were found improve functional recovery after I/R similarly to hearts submitted to IPC [6, 7]. In the current study however, no significant improvement in functional recovery could be detected after perfusion with any of the tested NO-donors (Table 1). These discrepancies could be due to differences in the experimental protocols and the analyzed parameters. Nonetheless, the above result suggests that NO^+^/NO^.^ can generate a cardioprotective effect when administered before prolonged ischemia and reperfusion. ONOO^-^ on the other hand was found to evoke a concentration dependent cytotoxic effect.

Oxidative damage is considered to be an important factor in cardiac damage after I/R and can result in the dysfunction and degradation of oxidatively modified proteins [53]. The MCRC catalytic antioxidant system is involved in the protection against oxidative stress-induced cell injury. In the present study, the components of the MCRC were analyzed to investigate the effects that different NO-donors might have on the antioxidant defense system. Msr activities, were significantly increased after treatment with 100 μM SNP and 10 μM SNAP, and suggest activation of the hearts’ antioxidant defense pathways (Table 2). Note that the measured Msr activities are independent of Txr, as they were performed in the presence of excess reducing agent (DTT). Increased Msr activities have been found to be associated with increased MsrA and MsrB expression levels [54, 55]. Detailed analyses revealed that the Msr activity was significantly increased immediately after perfusion with the NO-donor (Pre-Ischemia) and remained significantly elevated along the entire experiment. In line with a previous study by Picot *et al*., ischemia alone did not affect Msr activity [56]. Small decreases in Mrs activities of 37% and 24% were observed at the end of reperfusion in SHAM, I/R and 10 μM SNAP perfused hearts, respectively. It has been suggested that a decrease in Msr activity, after I/R, is not associated with a decrease in MsrA protein levels, but most likely caused by oxidative modifications of e.g. cysteine residues [56, 57]. Harmful oxidative modifications could explain the relatively low Msr activities in hearts exposed to I/R alone (Table 2). Even though no markers of oxidative stress (e.g. protein carbonyl) were investigated in the current study, the MCRC is known to play a key role in oxidative stress regulation. For example, there were greater increases in the levels of oxidized proteins (protein carbonyls) in tissues of *MsrA* null mice compared to wild type [29]. In addition, in old rats and in the brains of patients with Alzheimer’s disease there were decreases in MsrA activity that consequently lead to accumulations of carbonyl adducts in proteins [32, 58, 59].

Trx protein levels were dramatically increased after I/R (Table 3). These findings are in line with previous studies that found that Trx can be induced by oxidative stress [60, 61]. In the current study, perfusion with NO-donors were found to partially reverse the stimulatory effects of I/R on Trx protein expression levels. At the end of reperfusion, lower Trx levels were observed in all hearts when compared to I/R. These results mirror the observed increases in Mrs activities after NO-donor treatment, as measured at the end of reperfusion (Table 2). These findings correlate with previous studies and suggest that the induction of Trx is not linked directly to Msr activity, but is important for the function of e.g. Met(O) reduction [29]. This is not surprising as Trx has been found to be associated with other numerous cytoprotective effects including; regulation of transcription, regulating of apoptosis and co-cytokine activities [62]. In accordance with the reduction in Trx protein levels, at the end of reperfusion, 10 μM SNAP treated hearts also show a significantly reduction in TrxR protein levels.

Increasing Trx levels before I/R has been found to generate protection against I/R injury in a variety of studies [63–65]. In concurrence, the results presented in this study show that perfusion with 10 μM SNAP significantly increased Pre-Ischemic Trx protein levels which suggest an activation of the MCRC antioxidant defense system right before the damaging I/R. The abovementioned findings show that, depending on its bio-active redox form, NO can significantly affect various components of the MCRC and possibly aid in cardioprotection against oxidative damage.

Although not fully investigated here, previous studies have shown discrepancies between protein levels and Msr, Trx and TrxR activities. These differences were suggested to be associated with post translational oxidations and/or nitrosylations of amino acid residues, leading to either inactivation or activation of proteins activities [32, 66]. Bulvik *et al*. showed a reduction in TrxR activity in heart homogenates exposed to oxidative stress, whereas no differences in TrxR proteins levels were detected [32]. The fact that hearts treated with I/R alone displayed significant signs of myocardial damage, as shown by hemodynamic parameters and infarct size analyses, suggest an overloaded and/or impaired antioxidant defense. Possibly, the high Trx protein levels after I/R observed in this study are the results of an unsuccessful mechanism to compensate for the loss of Trx protein activity. A similar mechanism involving the MCRC system was previously observed in aging [32]. Furthermore, post translational S-nitrosylation of a regulatory Trx cysteine residue was found to be required for the increased activity and anti-apoptotic mechanisms in endothelial cells [66]. These observations suggest that exogenous NO-donors, increase Trx activity, without affecting protein expression levels, and so aid in the protection against the oxidative stress generated by I/R. The smaller infarct sizes, increased Msr activities, decreased Trx and TrxR proteins levels, all support this hypothesis.

Iron, an essential consistent of many macromolecules, is involved in energy production, respiration and metabolism. Excess ‘labile iron’ however, can enhance ROS-induced oxidative damage through the formation of hydroxyl radicals and shift the redox balance from cell survival to cell death [67, 68]. Because of its redox activity, most of the cellular iron is protein-bound by ferritin. Ferritin levels are post-transcriptionally regulated by IRP1 and IRP2. When iron is abundant, IRP1 and 2 dissociate from the ferritin mRNA, allowing its synthesis. After dissociation, IRP2 is targeted for proteasomal degradation, whereas IRP1 assembles an Fe-S cluster displaying CA activity [69, 70]. Targeted deletions have demonstrated that IRP2 is the main physiologic iron sensor in most organs [71–73]. In the current study, I/R was found to significantly increase CA activities compared to SHAM treated hearts (Table 4). Furthermore, the NO-donors 10 μM SIN-1 and 10 μM SNAP significantly increased CA activities compared to I/R treated hearts. The observed increase in CA activity after perfusion with 10 μM SNAP was also associated with increased levels of ferritin levels (Table 5). These observations suggest that the activation of ferritin synthesis is a result of the increased levels of intracellular iron, and the subsequent dissociations of IRP1 and IRP2 from the ferritin mRNA. Interestingly, 2 μM SIN-1 perfused hearts were associated with lower ferritin levels and a higher number of iron atoms per ferritin molecule (3156 ± 79) N_Fe_. The ferritin molecule can harbor up to 4,500 iron atoms [74]. Thus the impression is that the ferritin molecule reaches saturation levels. This finding could explain the increase in infarct size, observed in 2 μM SIN-1 perfused hearts and the inferior preservation of the MCRC system. The abovementioned specific effects of the different NO-donors on ferritin levels have previously been observed in *in vitro* studies and are most likely related to the different bio-active redox forms of NO [25, 75–77]. Detailed analyses of ferritin levels, revealed that perfusion with 10 μM SNAP caused a ~1.5-fold increase in ferritin levels. A significant ~2.5-fold increase was observed at the high-risk moment of the onset of reperfusion. This accumulation of ferritin can protect the heart from iron-mediated oxidative damage associated with I/R injury, and resembles the accumulation observed after IPC. Nonetheless, IPC was found to generate a more robust increase in ferritin levels of 4-7-fold [33, 41]. This increase was accompanied by a reduction in infarct size as well as a significant improvement in hemodynamic function. The regulatory capacities of NO are dose-dependent, thus increasing the SNAP concentration could possibly generate more profound cardioprotective effects [7]. Alternatively, it can be hypothesized that NO is sufficient but not a trigger for generating the strong protection observed by IPC [8, 45].

The abovementioned findings indicate that exogenous NO, like IPC, can regulate ferritin accumulation and is involved in the IBM of cardioprotection [33, 34]. The role of NO was found to depend on its concentration and the identity of its bio-active redox form. Future studies employing NO scavengers and/or NOS inhibitors, during the IPC phase, will need to be conducted to confirm the role of endogenous NO in the IBM of cardioprotection. The current literature is divided on the requirement of the biosynthesis of endogenous NO for the development of IPC [45]. It is yet unknown if NO will act as a trigger or mediator during IPC and the IBM. Likewise, the exact role of NO in the proteasomal pathway remains to be elucidated [34]. We hypothesize that NO is involved in generation of the ‘iron signal’, and the subsequent ferritin accumulation leading to cardioprotection, through either direct (release of labile iron from iron centers) or indirect (nitrosylations and/or nitrations) effects.
